# The radioactive ^103^Pd and ^109^Pd palladium bipyridyl–bisphosphonate complexes for radionuclide therapy of bone metastatic tumor cells†

**DOI:** 10.1039/d5ra02172c

**Published:** 2025-06-03

**Authors:** Geeva Prasanth Annamalaisamy, Monika Lyczko, Aleksander Bilewicz

**Affiliations:** a Center of Radiochemistry and Nuclear Chemistry, Institute of Nuclear Chemistry and Technology Dorodna 16 Warsaw 03-195 Poland

## Abstract

Two radioisotopes of palladium, ^103^Pd and ^109^Pd, are considered promising candidates for therapeutic applications because they emit Auger electrons, which are known for their effectiveness in targeting and destroying cancerous cells. We synthesized complexes of ^103^Pd and ^109^Pd with two bipyridyl and one alendronate molecule. The complexes demonstrated stability and a strong affinity for the surface of hydroxyapatite grains, the main mineral component of bones. Radioactive complexes show significantly higher cytotoxicity against human prostate (DU 145) and ovarian Her2 positive (SKOV-3) cancer cell lines compared to trastuzumab labeled with the Auger electron emitter ^125^I and cisplatin. The biological studies showed that both ^103^Pd, a pure Auger electron emitter, and ^109^Pd, which emits both beta and Auger electrons, demonstrate high cytotoxicity. Furthermore, it was observed that in the tested complexes, ^109m^Ag, a decay product of ^109^Pd, was released from the complex following the decay of ^109^Pd. In contrast, ^103m^Rh, a decay product of ^103^Pd, remained within the structure of the complex. The release of ^103m^Rh from the ^103^Pd complex is inhibited by the presence of delocalized electrons in the aromatic bipyridyl ligand. The concept of using a ^109^Pd/^109m^Ag and ^103^Pd/^103m^Rh generator encourages further exploration of this treatment strategy.

## Abstract 2

The radioactive ^103^Pd and ^109^Pd palladium bipyridyl–bisphosphonate complexes for radionuclide therapy of bone metastatic tumor cells were studied. The complexes demonstrated stability and a strong affinity for the main mineral component of bones.

## Graphical abstract

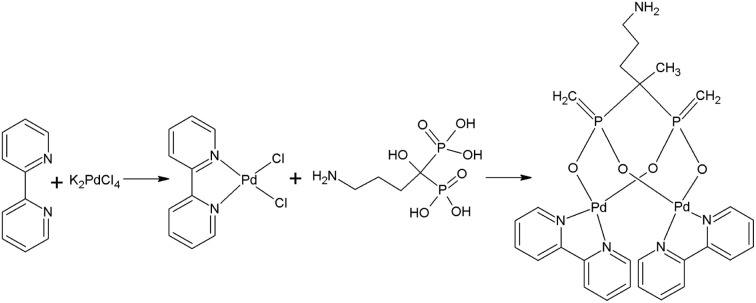

## 1. Introduction

The primary reason for treatment failure and the leading cause of death among cancer patients is the development of secondary tumours in distant organs or tissues far from the original cancer site, known as metastases. This spread is one of the most dangerous aspects of cancer, making it harder to treat and often associated with a poorer prognosis.^1^

After the liver and lungs, bones are the most susceptible site for cancer metastasis.^2^ This is because the bone microenvironment provides a fertile ground for various types of cancer cells.^3^ Bone metastases cause accelerated bone resorption leading to serious complications such as severe pain, spinal cord compression, and pathological fracture.^3^ Currently, bone metastases are treated symptomatically with pain management, systemic chemotherapy, radiotherapy, surgery, or a combination thereof. According to a review,^4^ the clinical administration of therapies for skeletal metastases is achieved by the use of painkillers, cytostatic chemotherapy, radiotherapy and especially by administering bisphosphonates and radiopharmaceuticals based on calcium analogues and phosphonates. The main clinical goals of these therapies are to relieve pain, improve quality of life, and reduce the risk of complications such as pathological fractures, spinal cord compression, and hypercalcemia (high levels of calcium in the blood). Unfortunately, it is very rarely possible to extend the time of survival.

Radionuclide therapy has been one of the most effective treatments for bone metastases for many years. For this purpose, α and β^−^ particle-emitting radiopharmaceuticals have been applied. Very good results are obtained in the treatment of pain as well as in the case of ^223^Ra (α emitter) prolongation of survival time in Castration-Resistant Prostate Cancer (CRPC) patients with bone metastases. Two classes of therapeutic bone-seeking radiopharmaceuticals are used in practice. Calcium-analogue radiopharmaceuticals such as ^89^SrCl_2_ and ^223^RaCl_2_, as well as polyphosphates of β^−^ emitters, mainly ^153^Sm, ^177^Lu, and ^188^Re are available. Radiation doses to bone metastases are limited due to the radiosensitivity of bone marrow, making it unsafe to increase them without risk of destroying the bone marrow.^5^ The low effectiveness of radiopharmaceuticals in treating bone cancer metastases is due to the necessity of using suboptimal doses. Hence, better therapeutic outcomes are achieved by using ^223^Ra, which emits short-range α particles. However, there can be serious complications during ^223^Ra therapy because the decay of ^223^Ra forms α-emitting decay products ^211^Pb and ^211^Bi, which can accumulate in healthy tissues.

However, it is expected that the use of Auger and conversion electron emitters can significantly improve the efficiency of polyphosphate-based radiopharmaceuticals, allowing not only the treatment of pain but also prolonging survival. The effectiveness of radiopharmaceuticals based on Auger and conversion electron emitters in treating bone metastases is due to the fact that the emitted energy is deposited in a very short range, on the micro and nanometre scale, without causing damage to the bone marrow. For the above reasons, Auger electron therapy is a promising treatment method, especially in the case of very small tumors, such as metastatic cancer.^6^ In the treatment of bone metastases, Auger and conversion electron emitters allow the use of very high therapeutic doses without damaging the bone marrow.^7^ An attempt was made to use ^117m^Sn for the treatment of bone cancer metastases.^8^ It emits monoenergetic conversion electrons, which allows for using large bone radiation doses without excessive radiation to the bone marrow. A high bone-to-marrow ratio of 11 was obtained. However, the ^117m^Sn-DTPA complex has minimal affinity for bone and tin as a p-block metal is easily trans chelated.^9^ Additionally, ^117m^Sn can only be obtained by irradiation with alpha particles in the reaction ^116^Cd(α,3n)^117m^Sn or in the inefficient reaction on fast neutrons ^117^Sn(n,n′γ)^117^Sn.
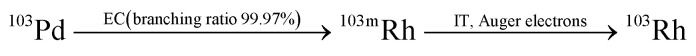


In the case of Auger electron emitters, only two papers on bisphosphonate complexes of Auger electron emitters have been published,^10,11^ and both concern ^195m^Pt. The authors synthesized a Pt^2+^ bisphosphonate (alendronate) complex in which two coordination sites of Pt^2+^ were occupied by the phosphonate group and two remained by ethylenediamine. Following attachment to bone cancer cells having a lower pH, the complex disintegrated and formed Pt (Et)Cl_2_, which diffused and penetrated the neoplastic tissue, intercalating into DNA in a similar way to cisplatin.^11^ Interestingly, this radiotherapeutic efficacy of ^195m^Pt-bisphosphonate was also much higher than ^223^Ra, since the 32-fold increased DNA damage caused by ^195m^Pt-bisphosphonate treatment significantly exceeded previously reported values for ^223^Ra treatment (only two- to threefold enhanced DNA damage).^10,11^ In our opinion, these studies open a new path in the treatment of cancerous bone metastases. Nevertheless, the problem in obtaining therapeutic activities of ^195m^Pt and also ^193m^Pt is a barrier to their widespread clinical application.^12^

The similar d^8^ electron configurations and high chemical similarity of Pd^2+^ and Pt^2+^ cations make palladium radionuclides interesting candidates for Auger electron therapy^13^ Additionally, numerous publications have confirmed the anti-cancer properties of Pd^2+^ cations and their complexes^14^ Therefore, the two palladium radionuclides, ^103^Pd (*t*_1/2_ = 16.99 d) and ^109^Pd (*t*_1/2_ = 13.59 h), can serve as excellent alternatives to ^195m^Pt. ^103^Pd is widely used in seed form for prostate brachytherapy because it emits X-ray radiation in the 20–23 keV range. The wide use of ^103^Pd in brachytherapy has led to the development of a method for its large-scale production. ^103^Pd can be produced in a reactor by irradiating a ^102^Pd target with neutrons or in a cyclotron by irradiating a natural ^103^Rh target with protons in p, n reaction. Although ^103^Pd emits only a few Auger electrons, ^103m^Rh produced by the decay of ^103^Pd is considered an attractive radionuclide for Auger electron therapy.^15,16^

Unfortunately, ^103m^Rh has a short half-life (*t*_½_ = 56.11 min), which is challenging and makes it impractical to prepare ^103m^Rh radiopharmaceuticals. However, it can be used in the form of an generator of ^103^Pd/^103m^Rh for targeted Auger electron therapy.

^109^Pd also has excellent potential for radionuclide therapy. ^109^Pd undergoes β^−^ decay (*β*_max_ = 1.12 MeV, 100% yield) to ^109m^Ag (*t*_½_ = 39.6 s). The formed metastable ^109m^Ag decays to stable isotope ^109^Ag, which is associated with the emission of an 88 keV photon (3.6%), which is subsequently followed by a cascade of emissions involving both conversion electrons and Auger electrons. Such properties enable its simultaneous application in both low- and high-LET internal radiation therapy. Recently published groundbreaking studies by Mueller *et al.*^17^ have demonstrated that the utilization of similar Auger and β^−^ electron emitter – ^161^Tb can lead to a significantly greater therapeutic effect compared to similar studies involving ^177^Lu.

In this study, we propose the utilization of ^103^Pd and ^109^Pd in the form of ^103^Pd/^103m^Rh and ^109^Pd/^109m^Ag generators for labeling of bisphosphonate in Auger electron therapy. Due to the limited availability of ^103^Pd, most studies were conducted using ^109^Pd. However, work with ^103^Pd will continue as we have secured supplies of ^103^Pd within the PRISMAP project in 2025. ^109^Pd can be obtained in reactor neutron irradiation. According to Das *et al.*,^18^ irradiation of enriched ^102^Pd (enrichment 98%) with neutrons with a flux of 3 × 10^13^ n cm^−2^ s^−1^ for 72 h gives radionuclide-pure ^109^Pd with a specific activity of 1.85 GBq mg^−1^. When a high-flux reactor (>10^15^ n cm^−2^ s^−1^) is used, the specific activity can increase to 40 GBq mg^−1^.

The ^103^Pd and ^109^Pd were utilized as mixed bipyridine–bisphosphonate complexes. Two papers on palladium alendronate compounds were published recently.^19,20^ In these publications, mixed complexes of Pd(ii) with alendronate and bipyridine or 1,10-phenanthroline were synthesized and studied. Their therapeutic effect on human prostate cancer (DU 145) and breast cancer (MDA-MB-231) cells was studied *in vitro* and compared with cisplatin. The results of the conducted studies indicate that the complexes exhibit greater cytotoxicity towards human prostate and breast cancer cell lines compared to normal human prostate and breast cell lines. The mechanism of the anti-cancer activity of these complexes has not been described in detail. Since Pd^2+^ has the same electron configuration (d^8^) as Pt^2+^, it can be assumed that the mechanism of the anti-tumor action of the Pd^2+^ phosphonate complexes should be similar to that of the described Pt^2+^ complexes^21^ The choice of NN co-ligands is aimed at highlighting DNA as a target. It is well known that metal complexes featuring extended planar aromatic ligands can reversibly bind to DNA by intercalating the planar aromatic portion between nucleobases.^19,22^ We chose the alendronate molecule as a bone-targeting ligand due to its high affinity for bone tissue. As previously mentioned, non-radioactive palladium diamine complexes with alendronate exhibited significant anti-tumor properties.^19,20^

In our publication, we also study the rejection of decay products ^103m^Rh and ^109m^Ag from ^103^Pd and ^109^Pd mixed aleodronian–bipyridine complexes. The release of Auger electron emitting decay products from the both palladium radioisotopes or their absence is crucial for the radionuclide therapy.

## 2. Materials and methods

### 2.1 Chemical reagents

Potassium tetrachloropalladium (K_2_PdCl_4_), 2,2′ bipyridyl (C_10_H_8_N_2_) were purchased from Sigma-Aldrich (St. Louis, MO, USA). Hydrochloric acid and sodium hydroxide were purchased from POCH (Gliwice, Poland). For cell and other biological studies phosphate-buffered saline (PBS), dimethyl sulfoxide (DMSO) was purchased from Sigma-Aldrich (St. Louis, MO, USA), Cell Titer® 96 Aqueous One solution reagent (MTS assay) from Promega (Mannheim, Germany). SKOV-3 and DU145 cell line bought from American Type Tissue culture collection (ATCC, Rockville, MD, USA) and were cultured in Mc Coy's and MEM-Eagles medium supplemented with 10% fetal bovine serum (FBS) and 1% penicillin–streptomycin (Beth Haemek, Isreal).

### 2.2 Radionuclides

^109^Pd was produced by irradiating 3 mg of natural palladium powder or 1 mg of >99% enriched ^108^Pd with a thermal neutron flux of 1.5 × 10^14^ n cm^−2^ s^−1^ for 7 hours at the Maria nuclear reactor in Poland. After a 3 h cooling time, the targets were dissolved in 200–400 μL of aqua regia (concentrated HNO_3_ : HCl–1 : 3) and then heated at 130 °C until complete evaporation. To convert nitrates to chlorides, the solid residue was dissolved in 0.1 M HCl three times (100 μL) and evaporated at 130 °C. Finally, the solid residue was dissolved in 1 mL of 6 M HCl to obtain H_2_PdCl_4_ solution.

When natural palladium (Pd) is irradiated with neutrons, it can produce ^111^Ag as an unwanted impurity in the reaction. ^110^Pd(n,γ)^111^Pd → ^111^Ag. It is crucial to remove it from the solution prior to application.^18^ The removal of ^111^Ag can be achieved by precipitating it as AgCl using the addition of AgNO_3_, following the modified procedure described by Das *et al.*^18^ Shortly, a total of 100 μL of a 0.1 M AgNO_3_ solution in 0.1 M HNO_3_ (equivalent to 20 mg mL^−1^) was added to a 1 mL solution of PdCl_4_^2−^ in 6 M HCl. After 2 minutes, the AgCl precipitate was collected by centrifuging the mixture at 4600 rpm for 5 minutes. The resulting supernatant was separated from the AgCl precipitate, and then was evaporated until completely dry. The procedure was repeated using deionized water, and finally, palladium was suspended in 100 μL of 0.1 M HCl. The diluted solutions obtained from the radiochemical processing of the irradiated target were analyzed using a High-Purity Germanium (HPGe) detector connected to a PC-based Multichannel Analyzer (MCA, Canberra). The 88 keV gamma peak, which has an abundance of 3.67%, emitted by ^109m^Ag, was utilized to determine the radioactivity of ^109^Pd. With an isotopically enriched ^108^Pd target, removal of silver is unnecessary due to the very low content of the ^111^Ag radionuclide.

^103^Pd was obtained as part of the European PRISMAP project from the Institute Laue–Langevin in Grenoble. The ^103^Pd was produced by 7 h irradiating 1.5 mg of an enriched ^102^Pd target (90.7%) in ILL High-Flux Reactor, with a neutron flux of >10^15^ n cm^2^ s^−1^. For the dissolution of the irradiated ^102^Pd target, the same procedure used for obtaining ^109^Pd was followed. After dissolution, we obtained 0.5 mL of solution with a concentration of 3.8 GBq mL^−1^ and with a specific activity of 1.3 GBq mg^−1^.

### 2.3 Instruments

Mass spectra were measured using the Q-TOF Premier mass spectrometer, Waters (Framingham, Massachusetts, USA) in the Institute of Biochemistry and Biophysics Polish Academy of Science.

FTIR spectra were recorded on the NICOLETE iS10 Thermo Scientific spectrometer (Waltham, Massachusetts, USA) in the 4000–400 cm^−1^ range using KBr pellets.

Radiochemical purity was assessed by Thin Layer Chromatography (TLC) using Watman Chromatographic paper 3MM. The chromatogram was developed by water/acetonitrile mixture (1 : 3 v/v) dried and the radioactivity distribution on the paper strip was measured by Cyclone® Plus Storage Phosphor System.

The identity studies of the radioactive ^109^Pd_2_(bpy)_2_ale complex (trace level) with the non-radioactive complex, synthesized from a macro amount of Pd and previously identified by mass spectroscopy, were performed using high performance liquid chromatography Merck Hitachi HPLC system (Tokyo, Japan) equipped with a reversed-phase C-18 column (Aeris Peptide 3.6μ XB-C18 150 × 4.60 mm) with water (A) and water/acetonitrile mixture (50/50) with 0.001 M EDTA as mobile phase. The following methods were used for complex separation (0–20 min 95% A to 5% B, 20–25 min 5% A to 95% B, 25–30 min 95% A to 5% B) with a flow rate of 1 mL min^−1^.

### 2.4 Synthesis of Pd_2_(bpy)_2_ale complex

The Pd_2_(bpy)_2_ale complex was prepared using a modified method based on the procedure reported by Cipriani *et al.*^20^ Briefly, aqueous solutions of 0.15 mmol of K_2_PdCl_4_ and 0.015 mmol of bipyridine ligand were added dropwise in equal molar proportions to the reaction mixture (total volume 5 mL) and stirred at room temperature for 1 h. In the meantime, molar excess of alendronate (0.30 mmol) and 0.30 mmol of triethylamine were prepared in 10 mL of aqueous solution. The mixture was added dropwise until the solution become clear brown and next was heated at 100 °C under reflux. The synthesis was maintained at the pH = 7. Once the solution was clear, it was concentrated to half its initial volume. Acetone was added to precipitate the product and the obtained precipitate was filtered and washed with diethyl ether and ethanol to remove unreacted bipyridine.

### 2.5 Synthesis of radioactive ^103^Pd_2_(bpy)_2_ale and ^109^Pd_2_(bpy)_2_ale complexes

To synthesize the radioactive complex, 420 MBq of ^109^Pd was added to the bipyridine (equal molar ratio) in 100 μL of water. After adjusting the pH to 7 using 0.1 M NaOH and HCl, the solution was sealed in an Eppendorf tube and left at room temperature for 1 hour. A 200 μL aqueous solution containing 100 times the excess of alendronate and triethylamine was added to the mixture dropwise while maintaining a pH of 7. The solution was then sealed again and heated to 60 °C for 1 h.

### 2.6 Stability of the complex

The stability of the complex was analyzed at 37 °C in human serum (HS) and PBS over 24 hours (∼2 half-lives of ^109^Pd). 50 μL of a radioactive complex was added to freshly prepared HS and PBS, and incubated at 37 °C for 24 hours. The stability was analyzed at specific time intervals using radio ITLC.

### 2.7 Release of ^109m^Ag from ^109^Pd_2_(bpy)_2_ale and ^103m^Rh from ^103^Pd_2_(bpy)_2_ale complexes

To study the release of ^109m^Ag from the ^109^Pd_2_(bpy)_2_ale complex, we employed an original procedure involving forming an insoluble AgCl precipitate, in which ^109m^AgCl is co-precipitated. We added 10 μL of the ^109^Pd_2_(bpy)_2_ale complex to a centrifuge tube containing 1 mL of NaCl solution, resulting in a radioactivity level of approximately 7.5 × 10^4^ cpm. After, to this solution, 100 μL of 0.1 M AgNO_3_ in 0.1 M HNO_3_ was added. The sample was mixed thoroughly, and the AgCl precipitate was separated from the solution using a centrifuge for 40 seconds. The supernatant was carefully pipetted away from the precipitate, and the AgCl was then dispersed in 1 mL of H_2_O. Activity measurements began 124 seconds after the precipitation of AgCl, which corresponds to three half-lives of ^109m^Ag, and were taken at 15 second intervals.

To investigate the release of ^103m^Rh from the ^103^Pd_2_(bpy)_2_ale complex, the ITLC technique was employed (refer to the stability section). The Cyclone® Plus Storage Phosphor System was utilized to measure the X-ray and low-energy γ radiation emitted by ^103^Pd and ^103m^Rh with high sensitivity.

### 2.8 Sorption on hydroxyapatite

Sorption on hydroxyapatite was performed according to the procedure described by Majkowska *et al.*^23^ Samples of hydroxyapatite weighing 50 mg, 75 mg, and 100 mg were added to 2 mL of saline and allowed to equilibrate for 1 hour. Following this, 50 μL of the radioactive ^109^Pd_2_(bpy)_2_ale complex was added, and the mixture was shaken at room temperature for 12 hours. The dispersion was centrifuged, and two aliquots of supernatant (2 × 500 μL) were taken to measure the activity using a scintillation gamma counter. The percentage sorption of ^109^Pd_2_(bpy)_2_ale complex was calculated using the formulae sorption (%) = (1 − *A*/*B*) × 100, where *A* is the average activity of the sample after equilibration and *B* is the initial activity of the solution.

### 2.9 Cytotoxicity studies

The MTS assay was employed to evaluate the ability of cells to proliferate. Tests were conducted on SKOV-3 (ovarian adenocarcinoma) and DU 145 (prostate cancer) cells. SKOV-3 and DU145 cells were maintained in McCoy's and MEM Eagle's media, respectively. Both cell types were seeded in 96-well plates at a density of 3 × 10^3^ cells per well, suspended in 100 μL of culture medium. After 24 hours of seeding, the ^109^/^103^Pd_2_(bpy)_2_ale complex with the desired activities (6 MBq mL^−1^, 12 MBq mL^−1^, 25 MBq mL^−1^, 50 MBq mL^−1^) was suspended in the medium and incubated for a period of 24 to 72 hours. Before adding 20 μL of MTS cells were washed with PBS and fresh medium was added (100 μL). Absorbance was measured at 490 nm.

### 2.10 DNA break (γH2AX)

γH2AX assay was performed to evaluate the double-strand break of DNA caused by the influence of ^109^Pd_2_(bpy)_2_ale complex. DU145 cells with density of 0.25 million cells per well were seeded in six well plates. Five sterile coverslips were placed in each well. After 24 h of incubation the medium was removed and treated with ^109^Pd_2_(bpy)_2_ale complex with two concentrations (25 MBq mL^−1^ and 6 MBq mL^−1^) and control cells. The cells were incubated for 4 h and 24 h. At proper time intervals the coverslips were transferred to 24 well plates (1 slip per well for each concentration). The slips were washed with PBS several times and 350 μL per well of primary antibody (anti-phospho-histone H2A.X (ser139)), clone JBW301 with dilution of 1 : 100 in blocking buffer and left at 4 °C overnight. Next day, wells were washed and treated with 250 μL per well of anti-mouse IgG(H + L) conjugated with CFTM633 and incubated for 2 h with time to time shaking at room temperature. Then the cells were washed with water several times and Hoechst 33258 was used for nuclei staining. The stained nuclei and breaks were analysed by FV-1000 confocal microscopy (Olympus corporation, Tokyo, Japan) with ex/em maxima: 630/650 nm for CF633 and ex/em maxima: 325/454 nm for Hoechst 33258. The images were then analysed by Fiji ImageJ.

## 3. Results and discussion

### 3.1 Synthesis of nonradioactive Pd_2_(bpy)_2_ale and radioactive ^103^Pd_2_(bpy)_2_ale and ^109^Pd_2_(bpy)_2_ale complexes

**Fig. 1:**
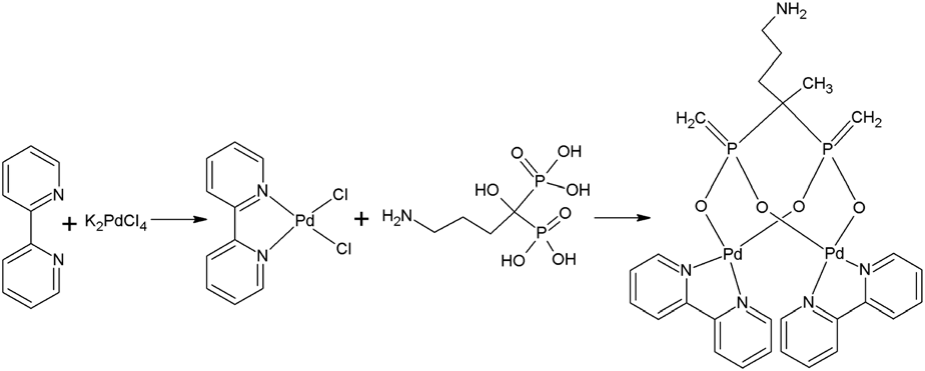
Synthesis of palladium complex with bipyridyl and alendronate ligands ^109^Pd_2_(bpy)_2_ale.

The synthesis procedure for the Pd alendronate bipyridyl mixed complexes is illustrated in Fig. 1. The initial step results in the formation of Pd(bpy)Cl_2_ complex, which is insoluble in water. The subsequent addition of alendronate leads to the formation of dinuclear species that contain one bisphosphonate for every two Pd(bpy) units. The complex obtained was yellow and soluble in water. Under similar synthetic conditions, Cipriani *et al.*^20^ also obtained a soluble in water yellow complex to which, based on elemental analysis, IR, and ^31^P NMR, assigned a structure in which two alendronate molecules bind to the Pd^2+^ cation *via* the terminal amine nitrogen. However, the authors did not perform identification by mass spectrometry. Since N-coordination is strongly favored under basic conditions, the synthesis was conducted at high pH. However, unlike Ciprani, we carried out the synthesis at pH = 7, where O coordination is preferred.

**Fig. 2:**
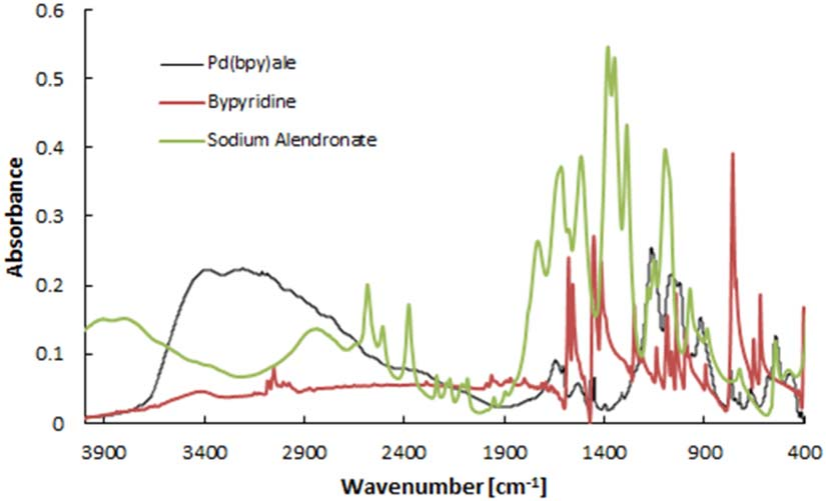
IR spectrum for non-radioactive Pd2(bpy)2ale complex, bipyridine, and sodium alendronate.

The recorded MS spectra unambiguously indicate the presence of the dinuclear complex with M^+^H^+^ 771.9360 *m*/*z* (calc. 771.9388) (Fig. S1†). In a similar synthetic procedure, such a complex was obtained and characterized in the work of Fathy *et al.*^19^ The IR spectrum recorded by us (Fig. 2) closely resembled the spectrum obtained by Fathy *et al.*, displaying characteristic band patterns that confirm the presence of the corresponding bipyridyl and bisphosphonate ligands in the synthesized palladium complex. The characteristic band between 1200 and 1600 cm^−1^ confirms the presence of bipyridyl ligand related to C=C and C=N vibrations. Phosphonate band region was observed in the region from 900–1300 cm^−1^. The most important band from *υ*(Pd–O) occurring at a wavenumber of 550 cm^−1^ confirms that the palladium in the complex is bonded to bisphosphonates through an oxygen atom. This band was not present in the studies of Ciprani *et al.*,^20^ indicating a difference in the structure of the obtained products.

The same procedure was used to synthesize the radioactive ^103^Pd_2_(bpy)_2_ale and ^109^Pd_2_(bpy)_2_ale complexes. The progress of the reaction was monitored by ITLC using water: acetonitrile (1 : 3 v/v), in which Pd_2_(bpy)_2_ale moved towards the solvent front (*R*_f_ = 0.7), while free PdCl_2_ remained at the origin (*R*_f_ = 0.1). The complexation efficiencies were over 80% for ^109^Pd and exceeded 85% for ^103^Pd. The highest yield was obtained at a temperature of 60 °C and a pH of 7.

HPLC chromatography was employed to compare the nonradioactive complexes with the synthesized ones at trace level radioactive ones. The chromatograms are presented in Fig. S2.† The presence of the same peak at 3.5 minutes in both the UV-Vis detector and the radio detector indicates that in both cases, the dinuclear Pd_2_(bpy)_2_ale complex presented in Fig. 1 is formed. The synthesized radioactive complexes maintain stability at 37 °C in human serum and PBS buffer for 24 hours, approximately two half-lives of ^109^Pd.

### 3.2 Liberation of ^109m^Ag from ^109^Pd_2_(bpy)_2_ale and ^103m^Rh from ^103^Pd_2_(bpy)_2_ale complexes

In nuclear medicine, when using *in vivo* generators, it is crucial to consider the behavior of the daughter radionuclide following radioactive decay. In our previously published papers, we studied liberation of ^109m^Ag from 5 nm ^109^Pd-PEG nanoparticles and the ^109^Pd-cyclam complex.^24^ Using the original procedure, we showed that while ^109m^Ag is completely released from the ^109^Pd-cyclam complex, in the case of 5 nm Pd nanoparticles ^109m^Ag remains in the nanoparticle.

The same procedure was used to study the release of ^109m^Ag from the ^109^Pd_2_(bpy)_2_ale complex. Fig. 5 illustrates the decay curve of ^109m^AgCl precipitated from the complex solution. Based on the decay curve it is found that the calculated half-life is similar to that of *t*_1/2_ 39 s of ^109m^Ag. Extrapolating the activity of the AgCl precipitate to time zero, we observed that the calculated activity of ^109m^Ag is close to the initial activity of ^109^Pd in the complex, evidencing the complete release of ^109m^Ag from the complex and coprecipitation as ^109m^AgCl. This aligns with the interpretation presented by Wang *et al.*,^25^ who hypothesized, based on the behaviour of ^166^Ho in the ^166^Dy/^166^Ho generator, that the loss of daughter radionuclides was due to their de-excitation through internal conversion. This process results in the emission of Auger electrons rather than the recoil energy associated with the emission of β^−^ particles and neutrinos. As a result the de-excited daughter radionuclides become highly charged, leading to electron uptake from surrounding chelator donor atoms.

When electron transfer occurs to highly charged atoms, the donor atoms in chelators become positively charged. This positive charge creates a repulsive force between the atoms, leading to breaking metal–ligand bonds. As a result, daughter radionuclides are released as free cations. In the case of the ^109^Pd–^109m^Ag system, this release also happens because formed Ag^+^ forms significantly weaker complexes than Pd^2+^.The longer half-life of ^103m^Rh (*t*_½_ = 56.11 min) compared to ^109m^Ag (*t*_½_ = 39 s) makes the release of Auger electron-emitting ^103m^Rh from ^103^Pd conjugates, or the absence of this release, significantly more important than that of ^109m^Ag.

**Fig. 3:**
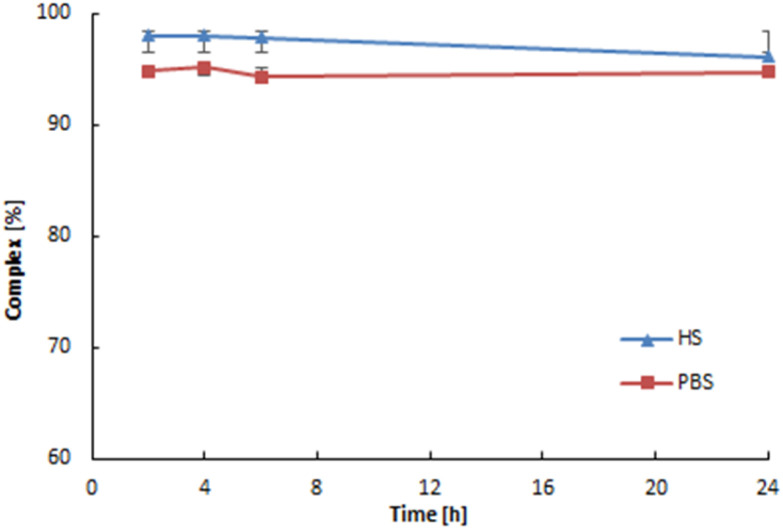
Stability of ^109^Pd_2_(bpy)_2_ale in human serum and PBS buffer.

**Fig. 4:**
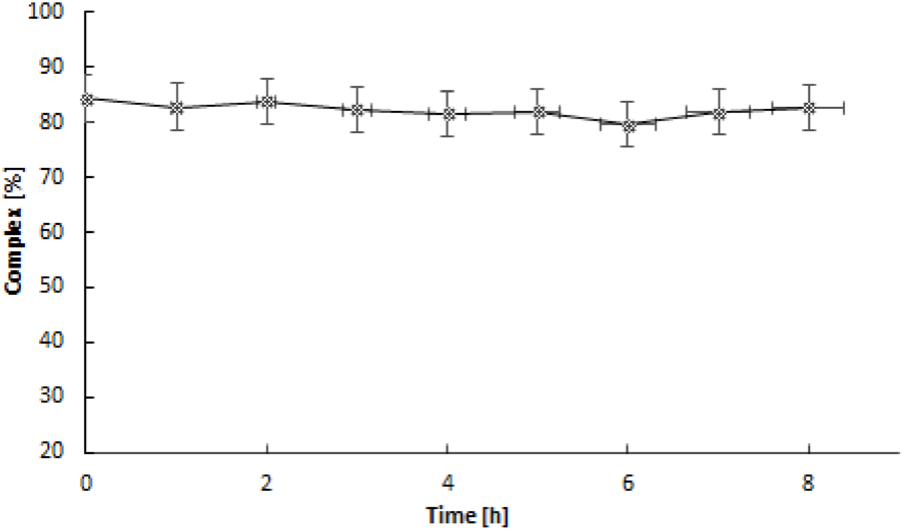
Retention study of Rh^3+^ in ^103^Pd_2_(bpy)_2_ale complex.

In our studies, we used the ITLC technique to investigate the release of ^103m^Rh from the ^103^Pd_2_(bpy)_2_ale complex. The ITLC recorded radioactivity on the strip using the Cyclone® Plus Storage Phosphor System, which measures X-ray radiation with high sensitivity. This allowed us to record the X-ray emitting positions on the strip emitted of both ^103^Pd and ^103m^Rh radionuclides. The results of stability studies conducted using ITLC in a water-acetonitrile system (Fig. 3). The liberation of ^103m^Rh from ^103^Pd complexes was previously examined during the development of the ^103sm^Rh radionuclide generator. In the studies of Jensen *et al.*,^26^ it was shown that after the decay of ^103^Pd, approximately 7% of ^103m^Rh is liberated from the macrocyclic [^103^Pd]16aneS4-*O*-octyl complexes. This phenomenon can be explained by Zeevaart and coworkers,^27,28^ who conducted detailed calculations of recoil energy associated with the emission of Auger electrons, photons, and neutrinos. They reported that the total energy generated is lower than the chemical bond energy in palladium–macrocycle complexes and is insufficient to allow the escape of particles from the complex. However, the highly positive charge of the Rh^*n*+^ cation after the emission of Auger electrons caused partial release of Rh^3+^ cations. The complete retention of Rh^3+^ in the ^103^Pd(bpy)ale complex observed in Fig. 4 is related to the presence of a bipyridyl ligand containing delocalized electrons. According to Nath *et al.*^29^ these ligands, such as for example heme, show the ability to quench the observed phenomenon known as the “Coulomb explosion” by transporting delocalized electrons to the metal core within time scales equivalent to the Auger cascade. It is also possible that after the decay of ^103^Pd, the resulting ^103m^Rh will be complexed only by bipyridyl ligand, forming the complex ^103m^Rh(bpy)^3+^. However, this cannot be confirmed by ITLC analysis.

**Fig. 5:**
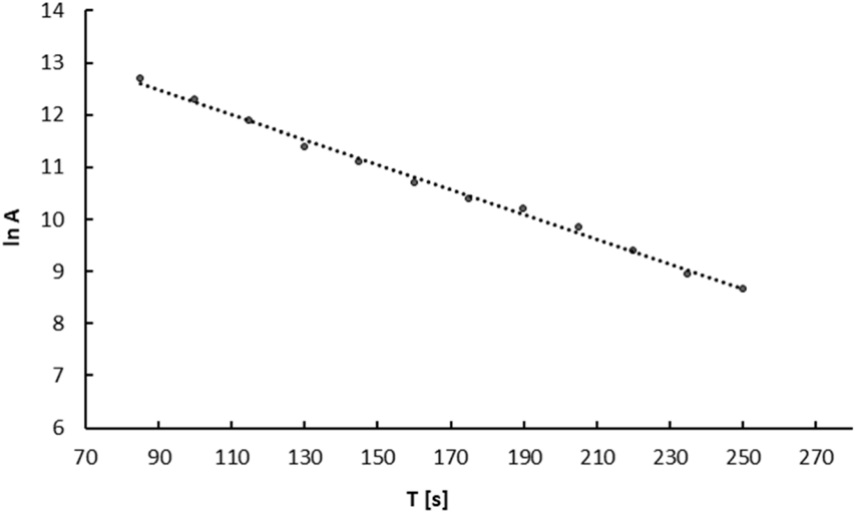
Radioactivity of AgCl precipitated from the ^109^Pd_2_(bpy)_2_ale complex solution. The measurement started 133 s after the addition of AgNO_3_. The radioactivity of the AgCl was measured successively at 15 second intervals. Activity in cpm.

### 3.3 Sorption of ^109^Pd_2_(bpy)_2_ale and ^103^Pd_2_(bpy)_2_ale on hydroxyapatite (HA)

The study of how effectively bone-seeking radiopharmaceuticals bind to bone matrix is a key indicator of the efficacy of any bone therapeutic agent or pain reliever. Hydroxyapatite (HA) particles, the fundamental chemical component of bone matrix, typically serve as the primary material for binding studies.^30^ HA is an ionic compound containing Ca^2+^, PO_4_^3−^, and OH^−^ ions on its surface. In bone cancer, the metabolism of bone is altered, leading to an increased uptake of calcium and phosphorus species, such as bisphosphonates. Since bisphosphonates have a strong affinity for Ca^2+^ ions, the primary mechanism for the adsorption of bisphosphonates is coordination to Ca^2+^ ions on the surface, as well as the potential substitution of surface phosphate anions.^31^ The adsorption is then described as bidentate coordination *via* two phosphonate oxygen atoms. In bisphosphonates complexes, the metal cation is coordinated by oxygen atoms from phosphonate. The same atoms are also responsible for the adsorption on the surface of HA. The complexes are kinetically labile and after adsorption on the HA surface, their transformation may occur. Thus, the adsorption of metal bisphosphonate complexes involves the dissociation and formation of the complexes, as well as the adsorption of both the complex species and its components (*i.e.*, the ligand and metal ion) separately.^32^ For example, the adsorption of Sn(ii) diphosphonate complex on hydroxyapatite occurs as the separate adsorption of Sn^2+^ and diphosphonate after dissociation of the complex.^33^

**Fig. 6:**
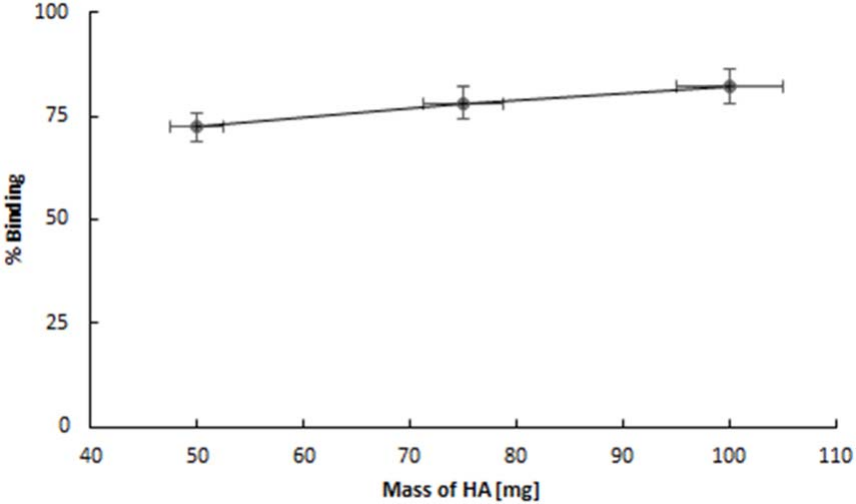
Adsorption of ^109^Pd_2_(bpy)_2_ale on HA grains.

**Fig. 7:**
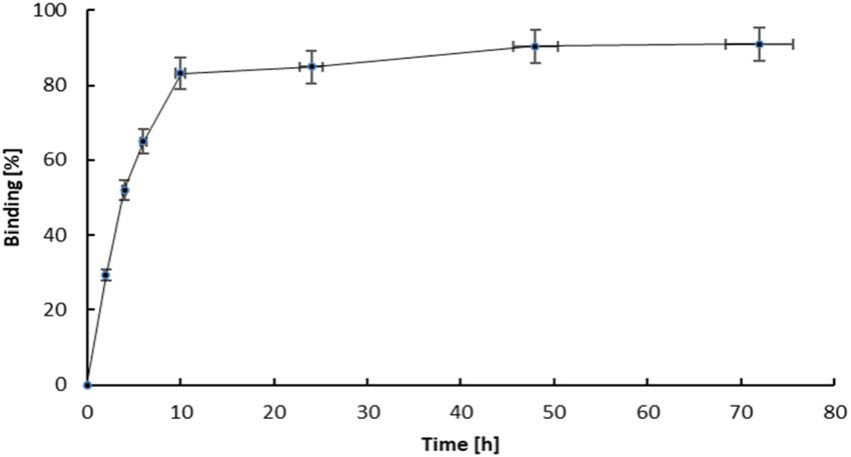
Adsorption kinetics of ^109^Pd_2_(bpy)_2_ale on HA grains.

The hydroxyapatite binding ability, represented as the percentage adsorption of ^109^Pd_2_(bpy)_2_ale zoledronates in relation to the amount of hydroxyapatite (HA), is illustrated in Fig. 6. The adsorption curve exhibits a profile similar to that observed for ^153^Sm-labelled alendronate; however, the percentage of binding is higher. Fig. 7 shown kinetic of adsorption of trace level ^109^Pd_2_(bpy)_2_ale complex on HAP samples. Pd_2_(bpy)_2_ale uptake onto HAP reached maximum after 72 h with 96% adsorption. The course of the curve is analogous to that observed for the non-radioactive (1 μM) complex.^19^

However, in contrast to the results of these studies, we did not observe the desorption of ^109^Pd species from early adsorbed ^109^Pd_2_(bpy)_2_ale on HAP. This could be probably due to the shorter duration of the experiment, due to the half-life of ^109^Pd of 13.59 h.

### 3.4 *In vitro* toxicity

**Fig. 8:**
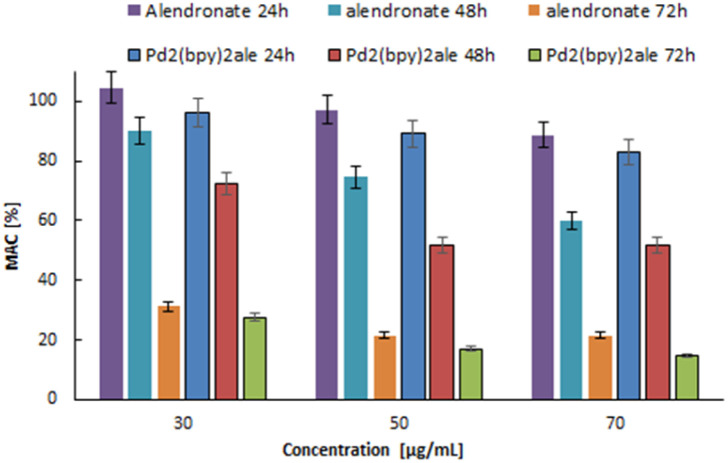
Metabolic viability of SKOV-3 cells after treatment with different concentrations of Pd_2_(bpy)_2_ale non-radioactive conjugates.

The cytotoxicity of nonradioactive Pd_2_(bpy)_2_ale and radioactive ^103^Pd_2_(bpy)_2_ale, ^109^Pd_2_(bpy)_2_ale was tested against cancer cells exhibiting the ability to form bone metastases. The viability of SKOV-3 cells overexpressing Her2 receptors, incubated with non-radioactive Pd_2_(bpy)_2_ale, was evaluated using the MTS assay. The Pd_2_(bpy)_2_ale was tested at concentrations ranging from 30 μg mL^−1^ (39 nmol mL^−1^) to 70 μg mL^−1^ (90 nmol mL^−1^). This study aimed to determine the cytotoxicity of the obtained bioconjugate toward the studied cells. The impact of non-radioactive Pd_2_(bpy)_2_ale on the viability of SKOV-3 cells is shown in Fig. 8. As the concentration of the Pd_2_(bpy)_2_ale increases, the viability of the cells gradually decreases. However, cell viability remains stable between concentrations of 50 and 70 μg mL^−1^. Additionally, in all concentrations examined, toxicity significantly increases with longer incubation times. This toxicity profile is similar to that reported by Fathy *et al.*^19^ for Pd_2_(bpy)_2_ale on MDA-MB-231 cells. The results also show that the cytotoxicity of the Pd_2_(bpy)_2_ale toward SKOV-3 cells is similar to that of cisplatin. Specifically, at a concentration of 20 μg mL^−1^, cisplatin achieves a cell survival rate of 25%.^34^ In comparison, the same level of cell survival with Pd_2_(bpy)_2_ale is reached at a concentration of 30 μg mL^−1^.

**Fig. 9:**
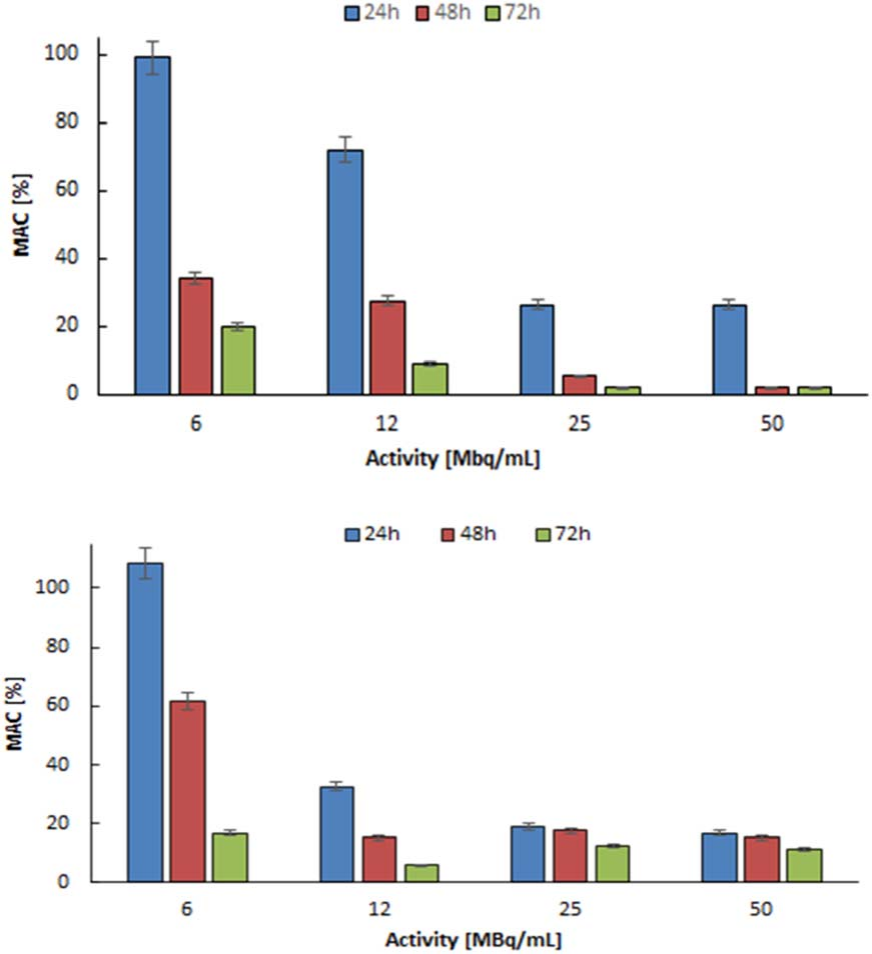
Metabolic viability of SKOV-3 cells after treatment with different concentrations of ^103^Pd_2_(bpy)_2_ale (up) and ^109^Pd_2_(bpy)_2_ale complexes (down).

The effects of different concentrations of radioactivity in ^103^Pd_2_(bpy)_2_ale and ^109^Pd_2_(bpy)_2_ale conjugates on the metabolic viability of SKOV-3 cells over 24, 48, and 72 hours of incubation are shown in Fig. 9. It is evident that the use of ^103^Pd_2_(bpy)_2_ale and ^109^Pd_2_(bpy)_2_ale radio conjugates significantly reduced the metabolic activity of SKOV-3 cells, with the reduction depending on the activity concentration and time interval after administration. An increase in the radioactivity of the radiobioconjugate progressively decreased mitochondrial activity, ultimately resulting in near-total cell death for an activity of 25 MBq mL^−1 103^Pd_2_(bpy)_2_ale after 48 h. Even at the lowest radioactivity, metabolic activity was below 30% after 72 hours. In the case of Pd_2_(bpy)_2_ale complex labelled with ^109^Pd, the cytotoxic effect is slightly smaller. This is primarily due to the shorter half-life of ^109^Pd, which is significant for longer incubation times. Also, the type of emitted radiation must be considered. The *in vivo* generator ^109^Pd/^109m^Ag emits β^−^ radiation as well as conversion and Auger electrons. In contrast, ^103^Pd/^103m^Rh emits only low-energy conversion and Auger electrons, although the number of electrons emitted by ^103^Pd/^103m^Rh is much higher.

When analysing the results presented in Fig. 9, it is important to note that the individual solutions of ^103^Pd_2_(bpy)_2_ale and ^109^Pd_2_(bpy)_2_ale used in the cytotoxicity tests were prepared by diluting initial solutions of 50 MBq mL^−1^ Pd_2_(bpy)_2_ale, which were labelled with carrier-added ^103^Pd and ^109^Pd. In the solution of the 50 MBq mL^−1^ Pd_2_(bpy)_2_ale complex labelled with carrier-added ^109^Pd, the concentration of the conjugate was 36.6 μg mL^−1^ and in the same activity of ^103^Pd_2_(bpy)_2_ale complex, the concentration of the conjugate was 63.9 μg mL^−1^. Consequently, the observed total cytotoxic effect consists of a chemo toxic effect caused by the non-radioactive conjugate (Fig. 8) and a radiotoxic effect from both ^103^Pd and ^109^Pd. Comparing the Fig. 8 and 9 we can see that the radiotoxic impact of the radio conjugates is much greater than the cytotoxic one. For SKOV-3, obtained IC_50_ values ^103^Pd_2_(bpy)_2_ale 18.86 MBq mL^−1^ at 24 h, 3.77 MBq mL^−1^ at 48 h and 2.233 MBq mL^−1^ at 72 h. In case of ^109^Pd_2_(bpy)_2_ale 10.62 MBq mL^−1^ at 24 h, 6.8 MBq mL^−1^ at 48 h and 0.1 MBq mL^−1^ at 72 h.

**Fig. 10:**
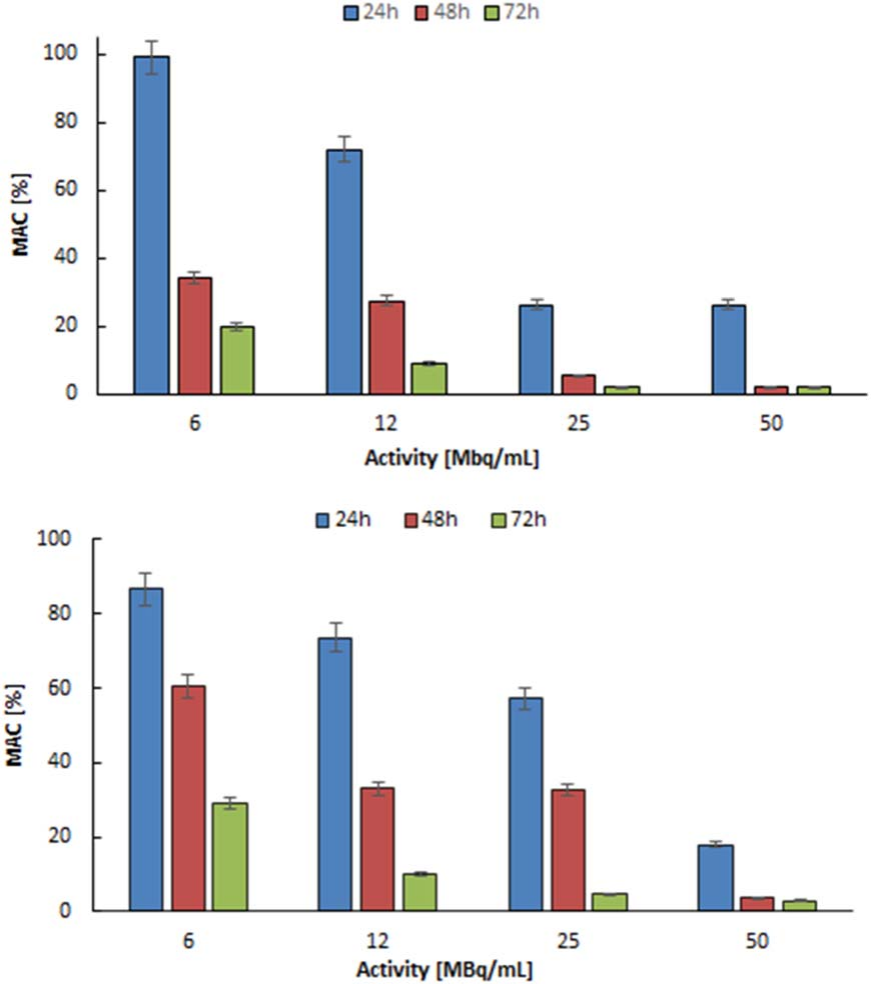
Metabolic viability of DU-145 cells after treatment with different concentrations of ^103^Pd_2_(bpy)_2_ale (up) and ^109^Pd_2_(bpy)_2_ale complexes (down).

Similar results were obtained in the cytotoxicity studies against DU-145 prostate cancer cells (Fig. 10). Like breast cancer, advanced-stage prostate cancer can also lead to numerous bone metastases. Increasing the radioactivity of the ^103^Pd_2_(bpy)_2_ale and ^109^Pd_2_(bpy)_2_ale caused a gradual reduction in mitochondrial activity, leading to almost complete cell death for 50 MBq mL^−1^ radioactivity for both radio conjugates. The impact of ^103^Pd and ^109^Pd labelled conjugates on DU-145 cells are nearly identical. As can be observed, the sensitivity for chemo and radiotherapeutics of SKOV-3 and DU-145 cells is similar. However, the toxicity of both radio conjugates is much higher than that of cisplatin administered in the same doses. It is even higher than in the case of the Auger electron emitter ^111^In-trastuzumab tested on Her2 positive SK-BR-3 cells.^35^

The strong anticancer properties of the non-radioactive Pd_2_(bpy)_2_ale complexes were explained by Fathy *et al.*^19^ due to their ability to bind to DNA. As an alternative to classical coordinate binding to DNA, metal complexes with planar aromatic ligands can also bind in a noncovalent π-stacking interaction, arising from the intercalation of a planar aromatic ring from the ligand between the base pairs of the DNA double helix.^36^ Such intercalating ligands are pyridine, 2,2′-bipyridine, 1,10-phenanthroline derivatives, and other similar ligands. Additionally, the increasing lipophilicity of the complexes makes them suitable for entering cells.^36^ This feature is further supported by the reported cytotoxicity of Pd^2+^ complexes with 2,2′-bipyridyl and 1,10-phenanthroline ligands, which show lower IC_50_ values than cisplatin in MTS assays of leukaemia cell line.^37^ For DU145, obtained IC50 values ^103^Pd_2_(bpy)_2_ale 20.31 MBq mL^−1^ at 24 h, 10.57 MBq mL^−1^ at 48 h and 4.48 MBq mL^−1^ at 72 h. In case of ^109^Pd_2_(bpy)_2_ale 24.68 MBq mL^−1^ at 24 h, 8.19 MBq mL^−1^ at 48 h and 3.45 MBq mL^−1^ at 72 h.

The possibility of intercalation of the Auger electron emitter complex into the double strand of DNA is crucial for the efficacy of the Auger electron therapy. Due to the range of Auger electrons being approximately in the range of the nanometre scale, the intercalation of ^103^Pd_2_(bpy)_2_ale and ^109^Pd_2_(bpy)_2_ale facilitates direct interaction of Auger electrons with DNA, as well as indirect interaction through reactive oxygen species formed by water radiolysis.^38^ As has been repeatedly stated, the short range of the emitted Auger electron cascade results in high levels of linear energy transfer (LET) 4–26 keV μm^−1^ allowing for double-stranded and irreparable DNA breakage.^39^ In the case of ^109^Pd, additionally, emitted β^−^ particles can interact with neighbouring cells through the so-called crossfire effect. Of course, the therapeutic effectiveness will be lower than Auger electrons due to the much smaller LET of β^−^ radiation.

### 3.5 DNA break

**Fig. 11:**
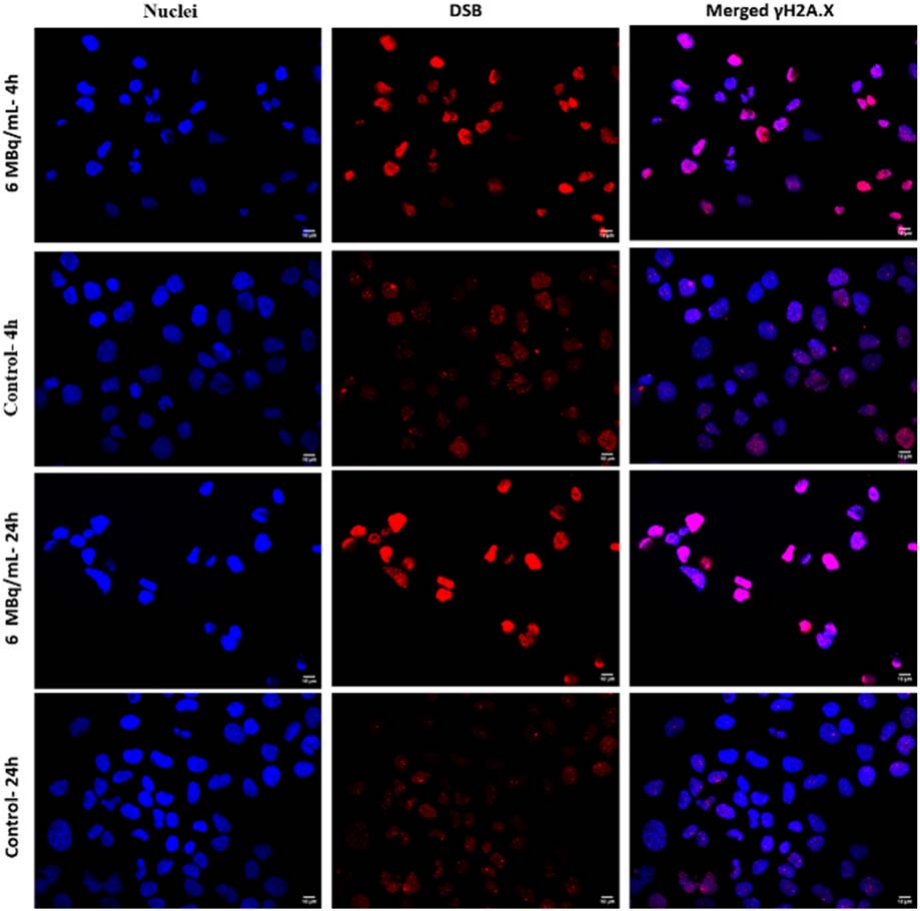
Microscopic images of nuclei (blue), γH2AX foci (red) and merging of both nuclei and γH2AX for the treated DU145 cells with ^109^Pd_2_(bpy)_2_ale after 4 h and 24 h of incubation.

Lethal and irreparable damage to genetic material is considered one of the most sought-after outcomes of the anticancer activity of radiopharmaceuticals.^40^ This damage may result either from the direct ionization of DNA by ionizing radiation or indirectly through interactions between DNA and reactive oxygen species (ROS) produced from water radiolysis. In our study, we examined the induction of DNA double-strand breaks (DSBs) following exposure to ^109^Pd_2_(bpy)_2_ale complex. Double-strand break experiment was performed in prostate cancer cell line DU145 treated with ^109^Pd_2_(bpy)_2_ale complex whose concentration was 25 MBq mL^−1^ and 6 MBq mL^−1^ (18.2 μg mL^−1^ and 4 μg mL^−1^). DNA double-strand breaks in DU145 cells are visualized with γH2AX foci after treatment with 6 MBq mL^−1 109^Pd_2_(bpy)_2_ale (Fig. 11).

**Fig. 12:**
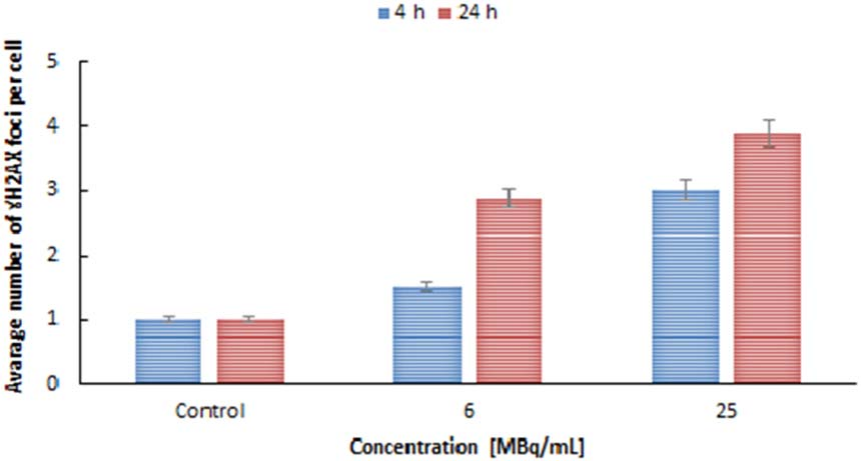
Analysis of γH2AX foci per cell with different time and concentration.

Quantification of these foci, represented as the integrated density of γH2AX foci per nucleus area is shown in Fig. 12. Cells with untreated doses showed average of about 1 double strand break up to 24 h. For 6 MBq mL^−1^ only 1.5 γH2AX foci was recorded after 4 h of treatment only slight difference from that of untreated ones. When comparing it to the longer incubation time say 24 h the number of γH2AX is in increasing pattern. Fig. 12 shows that signals of DNA damage are dose and time-dependent manners, which indicates the interaction between the high LET radiation exposure and DNA break formation. The presence of DSBs in the DU145 cells treated with ^109^Pd_2_(bpy)_2_ale is consistent with the cytotoxicity results shown in Fig. 9. In addition to DNA breaks, at higher concentration (25 MBq mL^−1^) even at 4 h of treatment there were decrease in cell count, potentially due to increased DNA damage weakens the cellular repair.

## 4. Conclusion

In summary, nonradioactive binuclear Pd_2_(bpy)_2_ale complexes was synthesized and their structures were evaluated on the basis of mass spectroscopy and infrared analysis. The same procedure was used to synthesize the radioactive ^103^Pd_2_(bpy)_2_ale and ^109^Pd_2_(bpy)_2_ale complexes. Comparing HPLC chromatographs of macro amount non-radioactive complex with those of synthesized in trace amounts radioactive complexes shows that the same complexes are formed in both cases. Using ITLC we found in the ^103^Pd_2_(bpy)_2_ale complex complete retention of the daughter isotope ^103m^Rh. In the case of the ^109^Pd complex, the situation is different. Due to the higher recoil energy and the chemical differences between Pd^2+^ and Ag^+^, we observe the complete release of ^109m^Ag. However, its *t*_½_ = 39 s is short, so there should be no significant displacement of radioactivity outside the targeting area. The tested Pd_2_(bpy)_2_ale complexes show high affinity to the surface of hydroxyapatite grains, which is the main mineral component of bone. Nonradioactive and radioactive complexes exhibit high cytotoxicity against the human prostate (DU 145) and ovarian Her2+ (SKOV-3) cancer cell lines, higher than trastuzumab labelled with Auger electron emitter ^125^I and also cisplatin. Both the radioactive ^103^Pd_2_(bpy)_2_ale and ^109^Pd_2_(bpy)_2_ale conjugates exhibit multimodal toxicity, emitting Auger electrons, and demonstrate chemotoxicity comparable to the commonly used chemotherapeutic cisplatin. Additionally, ^109^Pd, like the popular ^161^Tb, emits β^−^ radiation. However, the radiotoxic effects of Auger electrons become dominant due to the relatively high internalization of the radio bioconjugate into the cell nucleus, and expected intercalation to DNA. The results presented in this publication are preliminary and need to be confirmed through studies on induced bone metastases. However, in our opinion, these findings are interesting and promising, which is why we chose to publish them. We plan to conduct further studies in the near future in collaboration with a team specializing in *in vivo* preclinical studies.

## Data availability

The data supporting this article have been included in the ESI.†

## Author contributions

Conceptualization: A. B. and M. L. formal analysis: G. P. A., M. L. and A. B; funding acquisition: A. B.; investigation: G. P. A., M. L.; methodology: G. P. A., M. L. and A. B.; supervision: A. B. and M. L.; visualization: G. P. A., M. L.; writing—original draft: A. B., G. P. A., M. L.; writing—review and editing: M. L., G. P. A. and A. B. All authors have read and agreed to the published version of the manuscript.

## Conflicts of interest

The authors declare no conflicts of interest.

## Supplementary Material

RA-015-D5RA02172C-s001
