# Supplementary material for: The radioactive 103Pd and 109Pd palladium bipyridyl–bisphosphonate complexes for radionuclide therapy of bone metastatic tumor cells
Source: RSC Adv. 2025 Jun 3;15(23):18501–11. doi: 10.1039/d5ra02172c (PMC12132093; doi:10.1039/d5ra02172c)
Supplement: RA-015-D5RA02172C-s001 [file RA-015-D5RA02172C-s001.pdf]

Supplementary Information (SI) for RSC Advances.
This journal is © The Royal Society of Chemistry 2025

Supplementary information

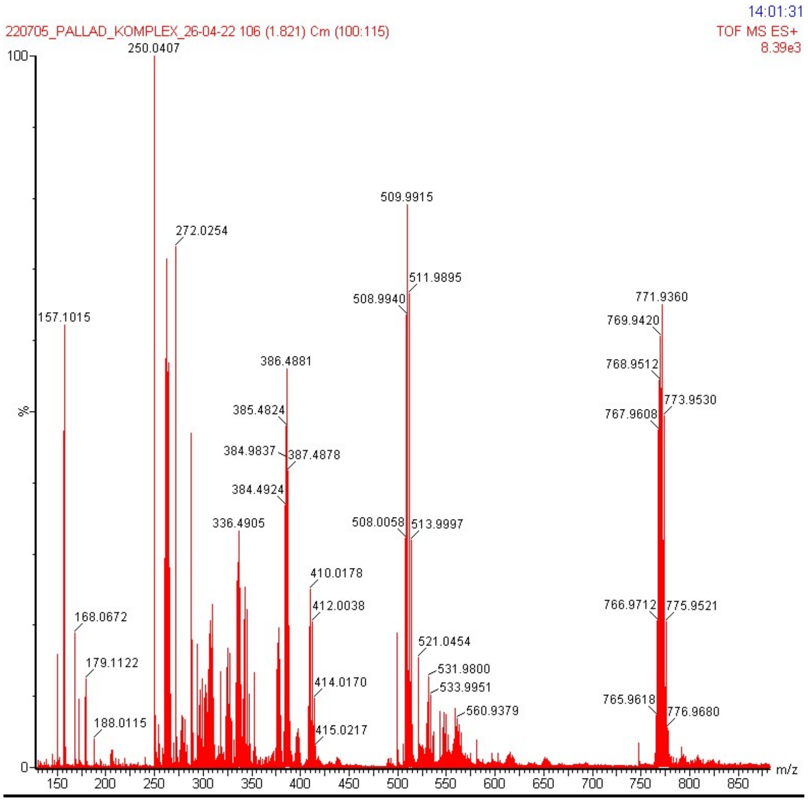

Figure 1S MS spectrum for $Pd_2(bpy)_2$ale

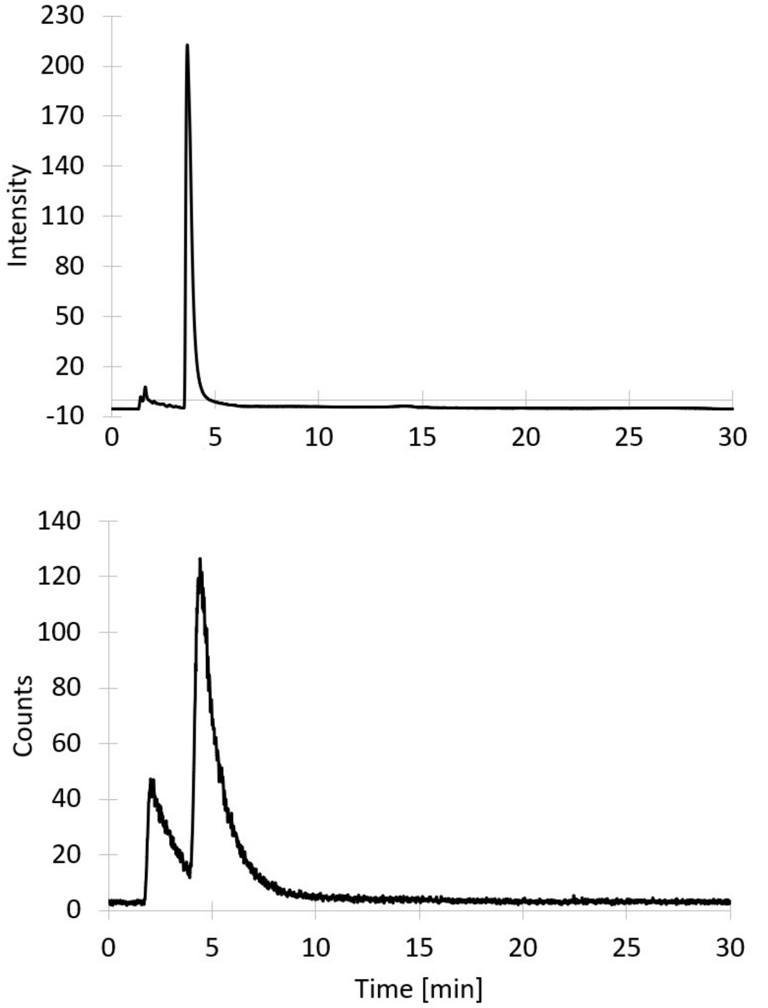

Figure 2S HPLC chromatogram for Pd2(bpy)2ale detected by UV detector at 300 nm, (up) and 109Pd2(bpy)2ale detected by [illegible] radiation detector (down)

Figure 3 Stability of $^{109}Pd_2(bpy)_2$ale in human serum and PBS buffer

HS				SD
Time	%complex		Average %complex	
2	97,9	98,2	98,05	0,212132
4	98,1	98	98,05	0,070711
6	98	97,8	97,9	0,141421
24	96,7	95,5	96,1	0,848528

PBS				SD
Time	%complex		Average %complex	
2	94,8	94,8	94,8	0
4	93,9	96,4	95,15	1,767767
6	94,8	93,9	94,35	0,636396
24	94,7	94,8	94,75	0,070711

Fig 4 Retention Study of Rh^{3+} in $^{103}Pd2(bpy)_2ale$ complex

%complex			Average %complex	SD
84	85,4	83,6	84,33333333	0,945163
84,3	83,8	80,2	82,76666667	2,236813
85,2	84,8	81,3	83,76666667	2,145538
83,4	83,2	80,2	82,26666667	1,792577
83,2	83,3	78,1	81,53333333	2,973774
82,7	83	80	81,9	1,652271
81,2	80,8	76,7	79,56666667	2,490649
84,2	82,9	78,2	81,76666667	3,156475
85,6	83,9	78,6	82,7	3,651027

Figure 5 Radioactivity of AgCl precipitated from the $^{109}Pd_2(bpy)_2ale$ complex solution. The measurement started 133 s after the addition of $AgNO_3$. The radioactivity of the AgCl was measured successively at 15-second intervals. Activity in cpm.

Measurement time	cpm	ln A
85	327894	12,70045
100	219363	12,29848
115	143838	11,87644
130	87199	11,37595
145	65650	11,09209
160	44567	10,70475
175	32360	10,38468
190	26902	10,19996
205	18815	9,84241
220	11954	9,388821
235	7645	8,941807
250	5779	8,661986

Figure 6 Adsorption of $^{109}Pd_2(bpy)_2$ale on HA grains

**Pd2(bpy)2ale**						
**Mass of Sorbent (mg)**	**Activity after 12hr**		**Total Activity**	**Blank Activity**	**% Sorption**	**SD**
50	337,2	342,2	679,5	2449	**72,25398122**	3,535534
75	264	272,7	536,7		**78,08493263**	6,151829
100	219	217	436		**82,19681503**	1,414214

Figure 7 Adsorption kinetics of $^{109}Pd_2(bpy)_2$ale on HA grains

**Pd2(bpy2)ale**							
**Time Interval**	**Activity of sample**		**Average activity**	**Blank Activity**		**Average Blank Activity**	**% Sorption**
0							0
2	22210	22030	22120	31280	31250	31265	**29,24996**
4	13320	13270	13295	27740	27600	27670	**51,951572**
6	8709	8696	8702,5	24890	24750	24820	**64,93755**
10	2061	2037	2049	12140	12160	12150	**83,135802**
24	1447	1432	1439,5	9488	9476	9482	**84,818604**
48	283,3	283,64	283,47	2929	2926	2927,5	**90,316994**
72	82,06	81,85	81,955	899,7	908,2	903,95	**90,93368**

Figure 8 Metabolic viability of SKOV-3 cells after treatment with different concentrations of $Pd_2(bpy)_2$ale non-radioactive conjugates

Pd2(bpy)2ale- SKOV 3					
**Concentration**	**70 µg/ml**	**50 µg/ml**	**30 µg/ml**	**Control**	**Background**
	0,976	1,075	1,156	1,219	0,135
**24hr**	1,071	1,093	1,142	1,179	0,13
	0,946	1,02	1,112	1,131	0,128
**average**	**0,997667**	**1,062667**	**1,136667**	**1,176333**	**0,131**
**SD**	0,065256	0,038031	0,02248		
**%MAC**	**82,90816**	**89,12628**	**96,20536**		
	0,868	0,905	1,029	1,638	0,129
**48hr**	0,868	0,848	1,19	1,543	0,127
	0,896	0,88	1,296	1,539	0,124
**average**	**0,877333**	**0,877667**	**1,171667**	**1,573333**	**0,12666667**
**SD**	0,016166	0,028572	0,134441		
**%MAC**	**51,8894**	**51,91244**	**72,23502**		
	0,441	0,478	0,634	2,13	0,133
**72hr**	0,402	0,449	0,697	2,117	0,131
	0,43	0,482	0,715	2,116	0,133
**average**	**0,424333**	**0,469667**	**0,682**	**2,121**	**0,13233333**
**SD**	0,020108	0,018009	0,042532		
**%MAC**	**14,6832**	**16,96279**	**27,63996**		

	**30**	**50**	**70**
**24h**	96,20536	89,12628	82,90816
**48h**	72,23502	51,91244	51,8894
**72h**	27,63996	16,96279	14,6832

Figure 9 Metabolic viability of SKOV-3 cells after treatment with different concentrations of $^{103}Pd_2(bpy)_2$ale (up) and $^{109}Pd_2(bpy)_2$ale complexes (down)

103Pd2(bpy)2ale- SKOV 3						
**Concentration**	**50 MBq/ml**	**25 MBq/ml**	**12MBq/ml**	**6MBq/ml**	**Control**	**Background**

24hr	0,697	0,549	1,294	1,723	1,724	0,137
	0,447	0,536	1,31	1,672	1,649	0,135
	0,497	0,556	1,194	1,675	1,745	0,134
**average**	**0,547**	**0,547**	**1,266**	**1,69**	**1,706**	**0,135333333**
**SD**	0,132287566	0,010148892	0,062864935	0,028618		
**%MAC**	**26,20967742**	**26,20967742**	**71,98641766**	**98,98132**		
48hr	0,165	0,292	0,759	1,084	2,37	0,138
	0,165	0,231	0,784	0,848	2,293	0,135
	0,192	0,227	0,646	0,677	2,247	0,134
**average**	**0,174**	**0,25**	**0,729666667**	**0,869667**	**2,303333**	**0,135666667**
**SD**	0,015588457	0,036428011	0,073527773	0,204363		
**%MAC**	**1,768414578**	**5,274488698**	**27,4027372**	**33,86129**		
72hr	0,186	0,201	0,381	0,535	2,844	0,136
	0,197	0,204	0,349	0,754	2,97	0,137
	0,189	0,178	0,412	0,722	2,703	0,138
**average**	**0,190666667**	**0,194333333**	**0,380666667**	**0,670333**	**2,839**	**0,137**
**SD**	0,005686241	0,014224392	0,031501323	0,118289		
**%MAC**	**1,986183074**	**2,121885023**	**9,01801135**	**19,73847**		

	**6**	**12**	**25**	**50**
**24h**	98,98132428	71,98641766	26,20968	26,20968
**48h**	33,86129479	27,4027372	5,274489	1,768415
**72h**	19,73846533	9,01801135	2,121885	1,986183

**109Pd2(bpy)2ale- SKOV 3**						
**Concentration**	**50 MBq/ml**	**25 MBq/ml**	**12MBq/ml**	**6MBq/ml**	**Control**	**Background**
24hr	0,255	0,307	0,472	1,067	1,029	0,134
	0,279	0,299	0,375	1,015	0,899	0,136
	0,3	0,284	0,394	1,093	1,033	0,138
**average**	**0,278**	**0,296666667**	**0,413666667**	**1,058333**	**0,987**	**0,136**
**SD**	0,02251666	0,011676187	0,051403632	0,039716		
**%MAC**	**16,68625147**	**18,87974931**	**32,62828045**	**108,3823**		
48hr	0,348	0,3	0,32	1,293	1,343	0,138
	0,387	0,45	0,369	1,033	1,569	0,143
	0,353	0,441	0,4	0,755	1,829	0,149
**average**	**0,362666667**	**0,397**	**0,363**	**1,027**	**1,580333**	**0,143333333**
**SD**	0,021221059	0,084124907	0,040336088	0,26905		
**%MAC**	**15,26327998**	**17,65251682**	**15,28647646**	**61,49385**		
72hr	0,443	0,506	0,273	0,664	2,623	0,133
	0,39	0,441	0,3	0,55	2,937	0,132
	0,493	0,446	0,298	0,531	2,869	0,136
**average**	**0,442**	**0,464333333**	**0,290333333**	**0,581667**	**2,809667**	**0,133666667**
**SD**	0,051507281	0,036170891	0,015044379	0,071933		

%MAC	11,5221724	12,35675137	5,854509218	16,74141		

	6	12	25	50
**24h**	108,3822953	32,62828045	18,87975	16,68625
**48h**	61,49385293	15,28647646	17,65252	15,26328
**72h**	16,74140508	5,854509218	12,35675	11,52217

Figure 10 Metabolic viability of DU-145 cells after treatment with different concentrations of $^{103}Pd_2(bpy)_2ale$ (up) and $^{109}Pd_2(bpy)_2ale$ complexes (down)

**103Pd2(bpy)2ale- DU145**						
**Concentration**	**50 MBq/ml**	**25 MBq/ml**	**12MBq/ml**	**6MBq/ml**	**Control**	**Background**
**24hr**	0,324	0,64	1,06	1,15	1,32	0,103
	0,328	0,674	1,166	1,207	1,467	0,105
	0,392	0,772	1,133	1,188	1,635	0,107
**average**	**0,348**	**0,695333333**	**1,119666667**	**1,181667**	**1,474**	**0,105**
**SD**	0,038157568	0,068537095	0,054243279	0,029023		
**%MAC**	**17,75018262**	**43,12149988**	**74,1173606**	**78,64621**		
**48hr**	0,131	0,417	1,039	1,214	1,796	0,109
	0,293	0,366	0,956	1,348	1,816	0,106
	0,147	0,433	0,928	1,242	1,867	0,106
**average**	**0,190333333**	**0,405333333**	**0,974333333**	**1,268**	**1,826333**	**0,107**
**SD**	0,089271123	0,034990475	0,057726366	0,070682		
**%MAC**	**4,84683986**	**17,3516867**	**50,44590927**	**67,52617**		
**72hr**	0,126	0,173	0,873	1,146	2,77	0,103
	0,184	0,268	0,764	1,147	2,67	0,106
	0,13	0,298	0,805	1,083	2,865	0,106
**average**	**0,146666667**	**0,246333333**	**0,814**	**1,125333**	**2,768333**	**0,105**
**SD**	0,032393415	0,065255907	0,055054518	0,036665		
**%MAC**	**1,564455569**	**5,306633292**	**26,62077597**	**38,31039**		

	6	12	25	50
**24h**	78,64621378	74,1173606	43,1215	17,75018
**48h**	67,52617294	50,44590927	17,35169	4,84684
**72h**	38,31038798	26,62077597	5,306633	1,564456

**109Pd2(bpy)2ale- DU145**						
**Concentration**	**50 MBq/ml**	**25 MBq/ml**	**12MBq/ml**	**6MBq/ml**	**Control**	**Background**
**24hr**	0,282	0,879	0,986	1,135	1,264	0,111

	0,345	0,542	1,049	1,168	1,347	0,111
	0,361	0,983	0,958	1,161	1,332	0,118
**average**	**0,329333333**	**0,801333333**	**0,997666667**	**1,154666667**	**1,314333**	**0,113333333**
**SD**	0,041765217	0,230530548	0,046608297	0,017387735		
**%MAC**	**17,98501249**	**57,28559534**	**73,63308354**	**86,70552318**		
**48hr**	0,179	0,878	0,741	1,398	1,879	0,104
	0,19	0,766	0,897	1,35	2,327	0,103
	0,198	0,837	0,852	1,559	2,713	0,105
**average**	**0,189**	**0,827**	**0,83**	**1,435666667**	**2,306333**	**0,104**
**SD**	0,009539392	0,056665686	0,080293213	0,10947298		
**%MAC**	**3,859542909**	**32,82881792**	**32,96503708**	**60,46617224**		
**72hr**	0,18	0,221	0,269	0,852	2,557	0,105
	0,168	0,201	0,372	0,72	2,318	0,109
	0,168	0,207	0,35	0,739	2,33	0,094
**average**	**0,172**	**0,209666667**	**0,330333333**	**0,770333333**	**2,401667**	**0,102666667**
**SD**	0,006928203	0,010263203	0,054243279	0,071360587		
**%MAC**	**3,015803973**	**4,654197477**	**9,902856314**	**29,0416123**		

	**6**	**12**	**25**	**50**
**24h**	86,70552318	73,63308354	57,28559534	17,98501
**48h**	60,46617224	32,96503708	32,82881792	3,859543
**72h**	29,0416123	9,902856314	4,654197477	3,015804

Figure 11 Microscopic images of Nuclei (Blue), γH2AX foci (red) and merging of both Nuclei and γH2AX for the treated DU145 cells with $^{109}Pd_2(bpy)_2$ale after 4h and 24h of incubation

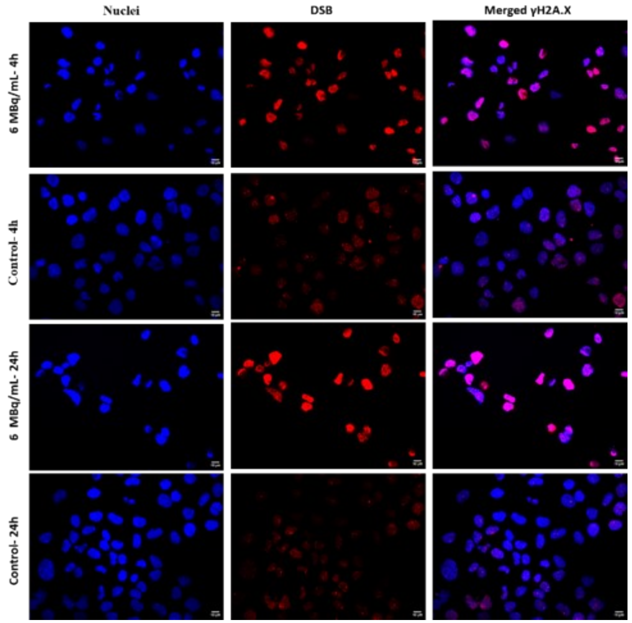

Figure 12 Analysis of γH2AX foci per cell with different time and concentration

Concentration	Particle Number	Integ. Density	InDen/PN	Mean	SD
6	84	132741,72	1580,258571	2013,375	423,2122
	113	176716,26	1563,860708		
	99	230136,75	2324,613636		
	114	300821,94	2638,788947		
	85	176542,38	2076,969176		
	118	223699,5	1895,758475		

Concentration	Particle Number		Integ. Density		InDen/PN	Mean	SD
25	28		128627,28		4593,831429	3976,516	567,8088
	28		112778,01		4027,786071		
	33		101050,02		3062,121818		
	34		144164,07		4240,119706		
	27		106885,53		3958,723333		

	PN	In Den	In Den/PN	Mean	SD
**Control 4 h**	268	426056,04	1589,761343	1322,69337	286,0412
	312	431729,55	1383,748558		
	360	363933,9	1010,9275		
	365	378221,13	1036,222274		
	295	469878,12	1592,807186		

	Concen.	Mean Den/PN	SD	DENS/Control	SD/Control
**4 h**	Control	1322,693372	286,04115	1	1
	6	2013,374919	423,212216	1,522178126	1,479550113
	25	3976,516471	567,808764	3,006378164	1,985059715

Concentration	Particle Number		Integ. Density		InDen/PN	Mean	SD
6	63		172574,1		2739,271429	3811,14	3061,083
	169		192113,46		1136,766036		
	44		407147,4		9253,35		

	334	365120,28	1093,174491		
	73	367718,22	5037,23589		
	102	367918,11	3607,040294		

Concentration	Particle Number	Integ. Density	InDen/PN	Mean	SD
25	3	27859,5	9286,5	5150,074	2571,255
	8	30560,31	3820,03875		
	11	28867,23	2624,293636		
	5	28836,09	5767,218		
	4	17009,28	4252,32		

	PN	In Den	In Den/PN	Mean	SD
**Control 24 h**	142	389077,3	2739,981	2177,04	1107,111
	214	389532,3	1820,245		
	308	358273,4	1163,225		
	116	443907,2	3826,786		
	394	525975,9	1334,964		

	Concen.	Mean Den/PN	SD	DENS/Control	SD/Control
**24 h**	Control	2177,040245	1107,111	1	1
	6	3811,13969	3061,083	2,881347839	10,70154613
	25	5150,074077	2571,255	3,893626584	8,989108747

Mean	Control	6 MBq/ mL	25 MBq/ mL
**4 h**	1	1,52	3,01

**24 h**	1,0	2,88134784	3,89
**SD**	**Control**	**6 MBq/ mL**	**25 MBq/ mL**
**4 h**	1	1,47955011	1,985059715
**24 h**	1	10,7015461	8,989108747
